# Long-term outcomes following transcatheter aortic valve implantation with the Portico self-expanding valve

**DOI:** 10.1007/s00392-023-02252-x

**Published:** 2023-06-30

**Authors:** Arturo Giordano, Silvia Mas-Peiro, Stephan Fichtlscherer, Andreas Schaefer, Martin Beyer, Francesco Maisano, Guido Ascione, Nicola Buzzatti, Rui Teles, João Brito, Francisco Albuquerque, Lars Sondergaard, Maarten Vanhaverbeke, Angelo Quagliana, Giuliano Costa, Marco Barbanti, Paolo Ferraro, Alberto Morello, Michele Cimmino, Michele Albanese, Martino Pepe, Luca Bardi, Salvatore Giordano, Antonio Cittadini, Nicola Corcione, Giuseppe Biondi-Zoccai

**Affiliations:** 1 Unità Operativa di Interventistica Cardiovascolare, Pineta Grande Hospital, Castel Volturno, Italy; 2 Unità Operativa di Emodinamica, Santa Lucia Hospital, San Giuseppe Vesuviano, Italy; 3 Department of Cardiology, Goethe University Hospital, Frankfurt, Germany; 4 Department of Cardiovascular Surgery, University Heart and Vascular Center Hamburg, Hamburg, Germany; 5 Department of Cardiac Surgery IRCCS, San Raffaele Scientific Institute, Milan, Italy; 6 Division of Cardiology, Hospital de Santa Cruz, Carnaxide, Portugal; 7 Heart Center, Rigshospitalet, Copenhagen, Denmark; 8 Division of Cardiology, A.O.U. Policlinico “G. Rodolico-San Marco”, Catania, Italy; 9 Division of Cardiology, Department of Emergency and Organ Transplantation, University of Bari, Bari, Italy; 10 Division of Cardiology, Department of Medical and Surgical Sciences, Magna Graecia University, Catanzaro, Italy; 11 Department of Medical and Translational Sciences, Federico II University of Naples, Naples, Italy; 12 Department of Medico-Surgical Sciences and Biotechnologies, Sapienza University of Rome, Latina, Italy; 13 Mediterranea Cardiocentro, Naples, Italy

**Keywords:** Aortic stenosis, Portico, Transcatheter aortic valve implantation, Transcatheter aortic valve replacement

## Abstract

**Aim:**

Transcatheter aortic valve implantation (TAVI) is a mainstay in the management of severe aortic valve stenosis in elderly patients, but there is uncertainty on their long-term effectiveness. We aimed to assess the long-term outcome of patients undergoing TAVI with the Portico valve.

**Methods:**

We retrospectively collected the data on patients in whom TAVI with Portico was attempted from 7 high-volume centres. Only patients theoretically eligible for 3 or more years of follow-up were included. Clinical outcomes, including death, stroke, myocardial infarction, reintervention for valve degeneration and hemodynamic valve performance were systematically assessed.

**Results:**

A total of 803 patients were included, with 504 (62.8%) women, mean age of 82 years, median EuroSCORE II of 3.1%, and 386 (48.1%) subjects at low/moderate risk. The median follow-up was 3.0 years (3.0; 4.0). The composite of death, stroke, myocardial infarction, and reintervention for valve degeneration occurred in 37.5% (95% confidence interval: 34.1–40.9%), with all-cause death in 35.1% (31.8–38.4%), stroke in 3.4% (1.3–3.4%), myocardial infarction in 1.0% (0.3–1.5%), and reintervention for valve degeneration in 1.1% (0.6–2.1%). The mean aortic valve gradient at follow-up was 8.1 ± 4.6 mmHg, and at least moderate aortic regurgitation was present in 9.1% (6.7–12.3%). Independent predictors of major adverse events or death were: peripheral artery disease, chronic obstructive pulmonary disease, estimated glomerular filtration rate, atrial fibrillation, prior pacemaker implantation, EuroSCORE II, and reduced left ventricular ejection fraction (all *p* < 0.05).

**Conclusions:**

Portico use is associated with favorable long-term clinical outcomes. Clinical outcomes were largely impacted by baseline risk factors and surgical risk.

**Supplementary Information:**

The online version contains supplementary material available at 10.1007/s00392-023-02252-x.

## Introduction

Transcatheter aortic valve implantation (TAVI) is currently the standard of care in Europe for patients aged 75 and higher with severe symptomic aortic valve stenosis [1–3]. Ongoing studies are evaluating whether patients younger than 75 years might also benefit from TAVI [3–6], and indeed in the US, patients are eligible for TAVI from 65 years. These advances towards lower risk patients increases the awareness of life time management of patients with severe aortic stenosis [7–10]. However, limited data is available on the long-term clinical and hemodynamic performance of these transcatheter heart valves.

Several devices for TAVI are currently available, with specific differences in terms of delivery system and THV design [11]. The Portico valve (Abbott Vascular, Santa Clara, CA, USA) is a self-expanding intra-annular device which is characterized by the ease of delivery and satisfactory early and mid-term outcomes, but limited evidence on long-term effectiveness [12–15].

We aimed at evaluating the performance of Portico beyond the early and mid-term timeframes, and conducted an international retrospective observational study focusing on long-term (≥ 3 years) clinical and imaging outcomes in patients undergoing TAVI with Portico.

## Methods

The current study is a spontaneous, not-for-profit, international, multicenter, observational and retrospective cohort study. Baseline clinical data, preprocedural transthoracic echocardiography with aortic valve gradients and procedural details were collected from 7 high-volume European TAVI centers (> 100 cases per year). The details on patients undergoing TAVI with the Portico valve were obtained by querying institutional databases, with ethical approval whenever appropriate. In particular, details on Italian centers were obtained from the ongoing the Registro Italiano GISE sull’impianto di Valvola Aortica Percutanea (RISPEVA) study, approved at Pineta Grande Hospital, Castel Volturno, Italy, as well as elsewhere (ClinicalTrials.gov Identifier: NCT02713932). Thus, all methods were carried out in accordance with relevant guidelines and regulations, all experimental protocols were approved by Pineta Grande Hospital, Castel Volturno, Italy, and informed consent was obtained from all subjects and/or their legal guardians. Notably, all patients receiving Portico at participating centers could be included, without any formal exclusion criteria, save for exclusion of individuals unwilling to allow anonymized data collection.

Briefly, we collected several baseline data including clinical history, comorbidities, and surgical risk scores. Baseline imaging data included transthoracic echocardiography details, such as left ventricular ejection fraction, aortic valve area, peak aortic valve gradient, mean aortic valve gradient, and aortic regurgitation. Patient-prosthesis mismatch, while not systematically sought, was approximated using a mean aortic valve gradient > 10 mmHg definition. Procedural features were systematically sought, including sheathless strategy, access site, predilation and postdilation. Clinical outcomes included all-cause death, cardiovascular death, stroke, myocardial infarction, major vascular complication, life-threatening bleeding, permanent pacemaker implantation, rehospitalization for cardiovascular causes, valve-related dysfunction requiring repeat procedure, endocarditis, leaflet thrombosis, and New York Heart Association class, applying whenever appropriate current Valve Academic Research Consortium 3 definitions [16]. The primary outcomes of interest were all-cause death and major adverse events (the composite of all-cause death, stroke, myocardial infarction, and reintervention for valve degeneration).

Descriptive analysis was based on the computing mean ± standard deviation for continuous variables, supplemented by median (1st quartile; 3rd quartile) whenever appropriate, and count (%) for categorical variables. Percentile bootstrapping techniques was used (1000 repetitions) to compute 95% confidence intervals of event rates. Failure curves were generated according to Kaplan and Meier, with hypothesis testing based on the log-rank test. Comparisons of interest for these log-rank tests included those based on the tertiles of age, and those based on the tertiles of surgical risk. We also identified independent predictors of death and major adverse events by conducting an iterative series of bivariate Cox proportional hazard analyses, and then including in the final model only predictors associated with *p* < 0.05 for the outcome of interest. The results of Cox proportional hazard analyses were reported as point estimate of hazard ratio, with corresponding 95% confidence intervals and p values. Competing risk analyses were performed for sensitivity purposes. Exploratory analyses focusing on Portico valve size were performed using unpaired Student’s *t* tests for continuous variables, and Fisher’s exact tests for dichotomous variables. In addition, we explored the impact of institutional learning phase (first 30 cases vs subsequent ones). Complete case analysis was used throughout. Statistical significance was set at the 2-tailed 0.05 level. Computations were performed with Stata 13 (StataCorp, College Station, TX, USA).

## Results

A total of 803 patients undergoing TAVI with Portico implantation were included (Table 1), with most procedures performed in 2016, 2017, and 2018 (Table 1S), and 3 centers contributing more than 100 cases each (Table 2S). The mean age was 82.2 ± 5.6 years, 504 (62.8%) were women, and 386 (48.1%) patients were at low surgical risk (EuroSCORE II < 3.0%), with median median EuroSCORE II of 3.1% (1^st^ quartile: 2.0%; 3^rd^ quartile: 5.8%), and median STSPROM score of 4.0% (2.8; 6.0%). Left ventricular systolic function was preserved in most patients and at least moderate aortic regurgiation was present in 162 (21.0%) subjects (Table 3S). Femoral access and local anesthesia were used in the vast majority of cases (Table 2) and predilation was used liberally (677 [84.6%]) cases. Device success was achieved in 778 (97.1%) procedures, with peri-procedural death occurring in 8 (1.0%), need for emergency surgery in 7 (0.9%), cardiac tamponade in 11 (1.4%), valve embolization in 14 (1.8%), and bailout TAVI-in-TAVI in 15 (1.9%).

**Table 1:** Baseline clinical features

Feature	Count or mean	% or standard deviation
Patients	803	–
Age (years)	82.2	5.6
Female	504	62.8%
Body mass index (kg/m^2^)	27.2	5.1
Hypertension	651	81.1%
Diabetic status		
Nondiabetic	577	71.9%
Noninsulin-dependent diabetic	156	19.4%
Insulin-dependent diabetic	70	8.7%
Peripheral artery disease	108	13.5%
Chronic obstructive pulmonary disease	121	15.1%
Prior stroke	61	7.6%
Estimated glomerular filtration rate	54.3	27.9
EuroSCORE II	5.1	6.0
STSPROM score	5.2	5.0
HASBLED score	2.7	0.9
Low surgical risk	386	48.1%
Frailty	319	39.7%
Prior myocardial infarction	95	11.8%
Prior percutaneous coronary intervention	236	29.4%
Prior coronary artery bypass grafting	77	9.6%
Prior aortic surgery	4	0.5%
Atrial fibrillation	198	24.7%
Prior pacemaker/implantable cardioverter–defibrillator	69	8.6%
Prior right internal mammary artery graft	1	0.1%
Prior left internal mammary artery graft	8	1.0%
New York Heart Association class		
I	10	1.3%
II	249	31.1%
III	473	59.1%
IV	68	8.5%
Aspirin	204	25.4%
P2Y12 inhibitors	84	10.5%
Antivitamin K agents	53	6.6%
Direct oral anticoagulants	102	12.7%
Urgent indication	25	3.1%

**Table 2:** Procedural features and in-hospital outcomes

Feature/outcome	Count or mean	% or standard deviation
Patients	803	–
Local anesthesia	750	93.4%
Access site		
Axillary	8	1.0%
Femoral	775	98.2%
Thoracic aorta	6	0.8%
Sheath size (French)	18.5	1.2
Sheathless	9	1.1%
Valve size		
23	19	5.4%
25	83	23.6%
27	110	31.3%
29	140	39.8%
Pacing site		
Right ventricle	797	99.2%
Left ventricle	6	0.8%
Predilation	677	84.6%
Postdilation	332	41.6%
Implantation of multiple devices	15	1.9%
Device success	778	97.1%
Mechanical cardiac support	4	0.5%
Fluoroscopy time (minutes)	19.6	12.1
Dose area product	11,065	11,448
Procedural time (minutes)	74.1	31.8
Contrast volume (mL)	139.7	76.9
Death	8	1.0%
Emergency surgery	7	0.9%
Cardiac tamponade	11	1.4%
Embolization	14	1.8%
Pacemaker dependency	109	14.6%
Complete heart block	75	13.0%
Total hospital stay (days)	9.5	8.4

**Fig. 1:**
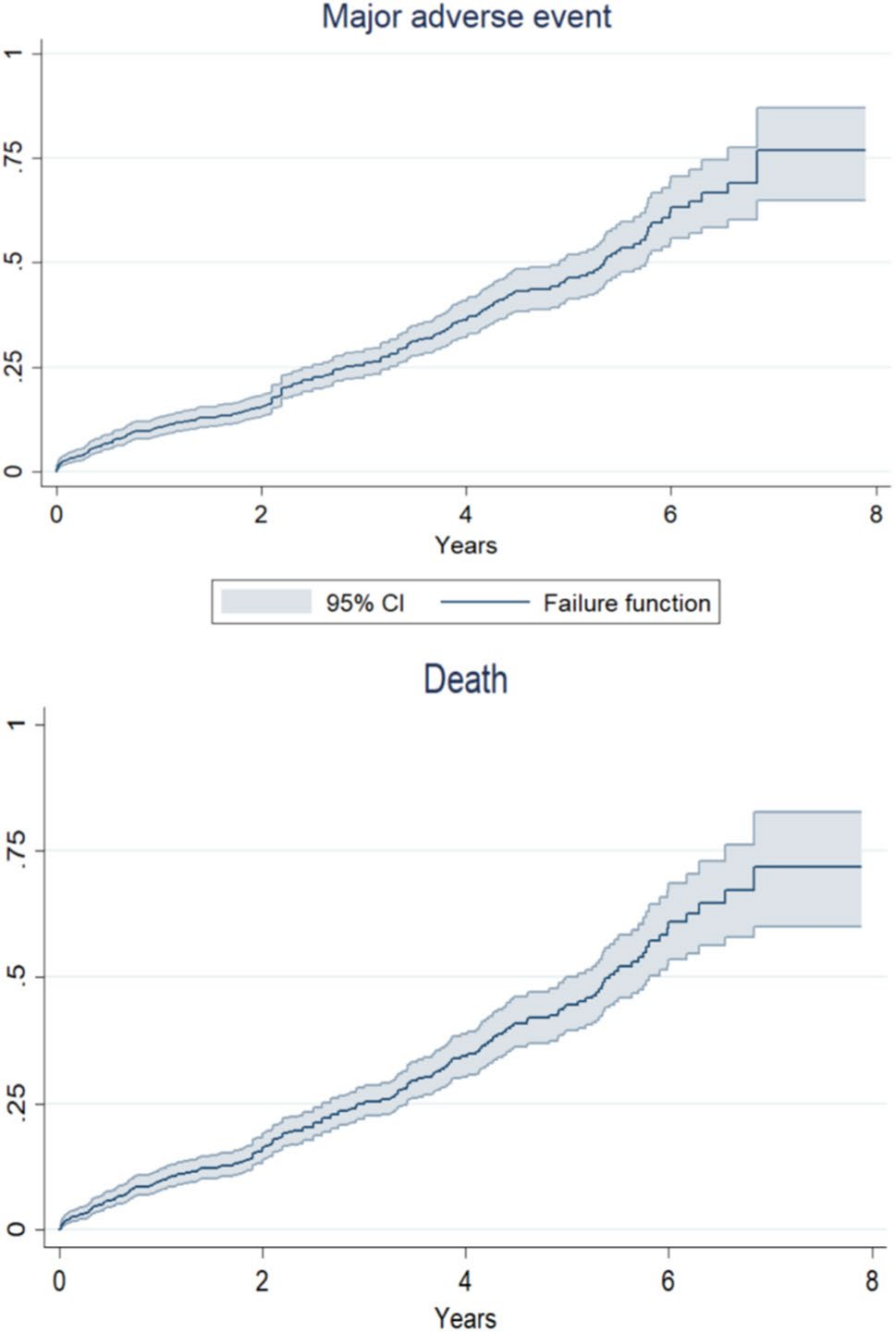
Overall incidence of major adverse event and death during long-term follow-up after transcatheter aortic valve implantation with the Portico valve

After a minimum follow-up of 3 years (median 3.0 years [3.0; 4.0]), major adverse events occurred in 301 (37.5% [95% confidence interval: 34.1%; 40.9%]) patients (Fig. 1). Death was the most common adverse event (35.1% [31.8%; 38.4%]) (Table 3; Fig. 1S). Although cardiovascular death was more frequent in the first 2 years after the procedure, noncardiovascular death occurred more frequently thereafter (Table 4S). The major vascular complications occurred in 40 patients (8.0% [5.6%; 10.3%]), permanent pacemaker implantation rate was 23.1% [19.4%; 26.7%], reintervention for valve degenaration was necessary in 9 (1.1% [0.6%; 2.8%]) cases, and, finally, 4 cases of endocarditis were reported, for an incidence of 0.5% (0.0%; 1.0%).

**Table 3:** Long-term outcomes

Feature/outcome	Count or mean	% or standard deviation
Patients	803	–
Events up to 1 month of follow-up		
Major adverse event^a^	38	4.7%
Death	31	3.9%
Cardiovascular death	25	3.1%
Stroke	11	1.4%
Myocardial infarction	0	–
Major vascular complication	9	1.1%
Life-threatening bleeding	8	1.0%
Permanent pacemaker implantation	81	10.1%
Rehospitalization for cardiovascular causes	2	0.2%
Valve-related dysfunction requiring repeat procedure	2	0.2%
Bioprosthetic valve failure	31	3.9%
Endocarditis	0	–
Follow-up (years)	3.1	1.5
Cumulative events		
Major adverse event^a^	301	37.5%
Death	282	35.1%
Cardiovascular death	190	23.7%
Stroke	17	2.1%
Myocardial infarction	5	0.6%
Major vascular complication	40	5.0%
Life-threatening bleeding	18	2.2%
Permanent pacemaker implantation	126	15.7%
Rehospitalization for cardiovascular causes	72	9.0%
Valve-related dysfunction requiring repeat procedure	9	1.1%
Bioprosthetic valve failure	284	35.4%
Endocarditis	4	0.5%
New York Heart Association class		
I	99	30.6%
II	154	47.5%
III	68	21.0%
IV	3	0.9%

^a^Death, stroke, myocardial infarction, and reintervention for valve degeneration

Long-term hemodynamic performance was assessed using echocardiography after a median follow-up of 3.1 years (2.0; 4.8). Mean aortic valve gradient at follow-up was 8.1 ± 4.6 mmHg, and at least moderate aortic regurgitation was present in 9.1% (6.7%; 12.3%) (Table 5S). Bioprosthetic valve failure, i.e. the composite of death, reintervention, and severe aortic regurgitation, occurred in 35.4% (32.0%; 38.8%). Mild-to-moderate or moderate aortic regurgitation at discharge was stable over time, with no case of worsening and improvement in most patients. Notably, smaller devices were associated with higher gradients, more patient-prosthesis mismatch, less regurgitation, and more adverse outcomes, even if this latter finding was largely due to worse risk profile (Tables 6S, 7S, and 8S).

**Fig. 2:**
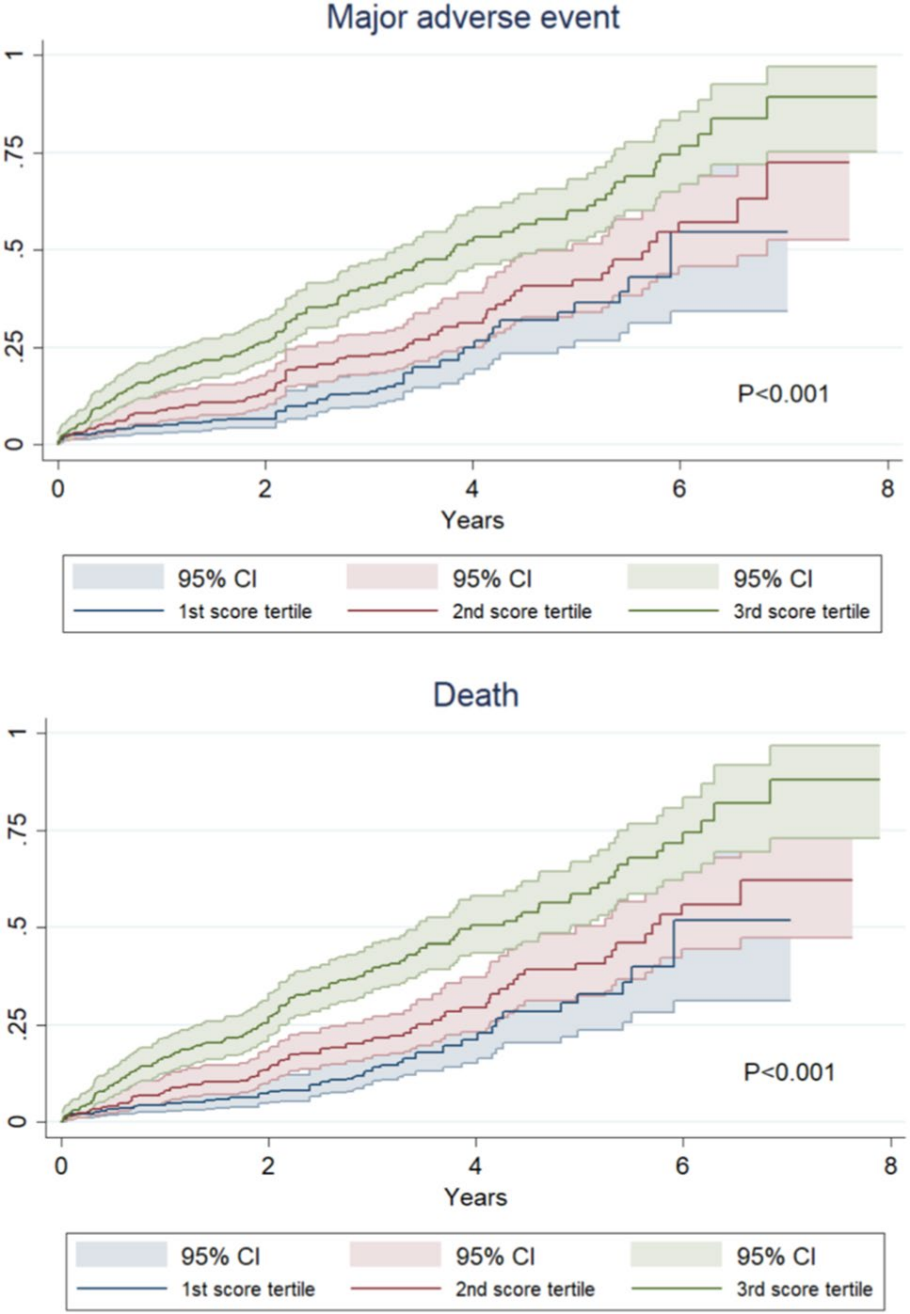
Incidence of major adverse event and death during long-term follow-up after transcatheter aortic valve implantation with the Portico valve according to tertiles of surgical risk (tertile 1: ≤ 2.30%; tertile 2: 2.31–4.54%; tertile 3: ≥ 4.55%)

**Fig. 3:**
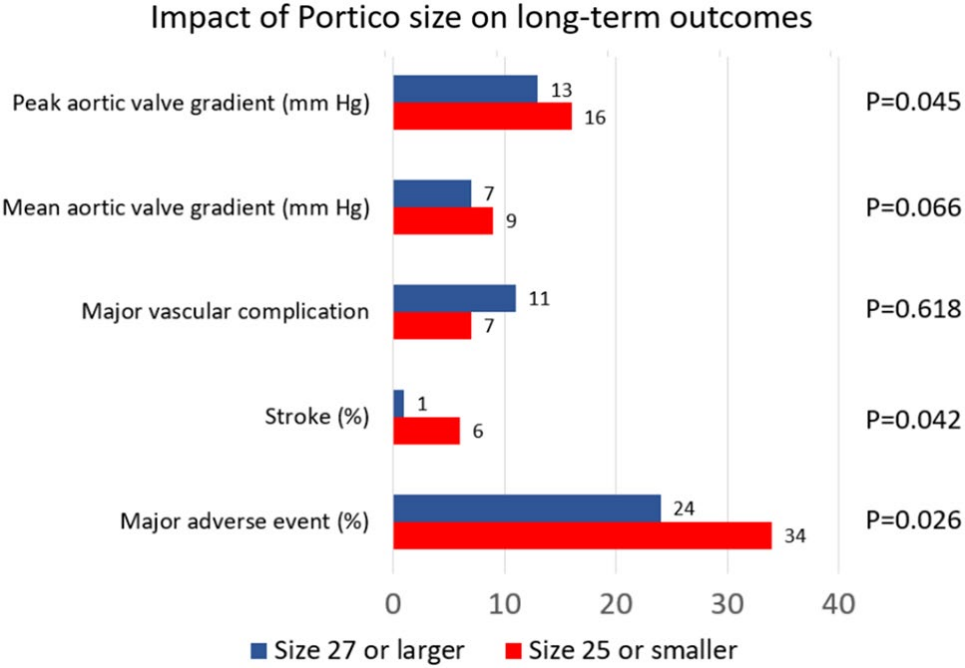
Exploratory analysis appraising the impact of valve size on long-term outcomes after transcatheter aortic valve implantation with the Portico valve

Clinical outcomes were not significantly impacted by age (Fig. 2S), but surgical risk was a significant bivariate predictor of events (Fig. 2). Further exploratory bivariate analysis assessing the impact of the device size suggested that TAVI with Portico valve size 25 mm or smaller was associated with an increase in risk of major adverse events (*p* = 0.026), stroke (*p* = 0.042), and higher peak aortic valve gradient (*p* = 0.045) (Table 8S; Fig. 3), but these findings were not confirmed at multivariable analysis. Indeed, the baseline differences in patient risk according to device size were most likely the cause of such discrepancies in event rates (e.g. EuroSCORE II was 4.8 ± 0.3 in patients receiving smaller devices in comparison to 4.0 ± 0.2 in those receiving larger devices, *p* = 0.044).

Indeed, at multivariable Cox proportional hazard analyses for major adverse events or death (Table 9S), peripheral artery disease, chronic obstructive pulmonary disease, estimated glomerular filtration rate, atrial fibrillation, prior pacemaker/implantable cardioverter defibrillator implantation, EuroSCORE II, and left ventricular ejection fraction were significant independent predictors (all *p* < 0.05). Competing risk analyses were consistent with Kaplan–Meier analyses, and yearly event rates appeared steady (Table 10S).

## Discussion

Transcatheter aortic valve implantation has seen a momentous expansion since the first human case description by Cribier et al. in [17]. The success of TAVI rests on many aspects, including refinements in patient selection, preprocedural imaging, devices, ancillary equipment (e.g. delivery systems), accompanying techniques (e.g., transaxillary access), and concomitant medical therapy [3]. Developments in devices have been quite substantial, and range from improvements of first-generation devices to the advent of completely original second-generation devices, such as Portico.

### Main findings and implications

Indeed, we hereby report, for the first time, the long-term clinical and echocardiographic follow-up of the self-expanding Portico TAVI valve in a multicenter international retrospective registry. We found that the Portico valve had reasonably favorable clinical outcomes, despite a non-negligible incidence of all-cause death over time, mainly related to an increased baseline risk profile [18–20].

Several devices for TAVI are now available, ranging from balloon-expandable devices to self-expandable devices with supra- or intra-annular leaflet implantation [21]. Irrespective of the chosen device, TAVI has clearly demonstrated its superiority to conservative management (with or without balloon aortic valvuloplasty) in patients who are not eligible for cardiac surgery due to increased operative risk [22]. Furthermore, TAVI has a favorable risk–benefit and cost–benefit profile in several other settings, including individuals with relatively low, moderate or increased but not prohibitive surgical risk [7, 8, 23]. However, the push towards use of TAVI in lower risk patients requires attentive scrutiny to long-term outcomes, ranging from cardiovascular events to specific valve-related features, including leaflet thrombosis [24].

### Focus on Portico

The Portico valve has been introduced into clinical practice several years ago, and it has been refined over the years to improve deliverability and mitigate the risk of paravalvular leak [25]. Indeed, it is now considered a valid alternative to other established devices for TAVI, such as Sapien (Edwards Lifesciences, Irvine, CA, USA), and Evolut (Medtronic, Minneapolis, MN, USA) [26]. While comparative studies report similar effectiveness for most TAVI devices [27], long-term studies on Portico are lacking. Indeed, comparison of outcomes provided by pivotal randomized trials, while always challenging, suggest that Portico can provide favorable early clinical results [3]. The same applies to comparative observational studies, such as the 1,976-patient registry sponsored by the Italian Society of Invasive Cardiology and focusing on 1-month outcomes, which showed similar rates of death (ranging from 1.5 to 2.7%), myocardial infarction (0–0.4%), stroke (0.4–2.7%) with Acurate (Boston Scientific, Natick, MA, USA), Evolut (Medtronic, Minneapolis, MN, USA), Lotus (Boston Scientific, Portico, and Sapien3 (Edwards Lifesciences, Irvine, CA, USA), despite significant differences for vascular complications, apparently favoring Lotus and Portico [28]. Similar results were obtained at 12-month follow-up from the same registry [27]. Additional sobering findings on Portico were previously reported from our group, in a study including 114 patients followed for a mean of 15 months) [14].

### Implications of the present study

In light of the current lack of long-term real-world data on Portico, the present results thus provide further evidence that Portico can be considered a promising TAVI device for patients with severe aortic stenosis who are not ideal candidate for cardiac surgery. The high event rates accrued during follow-up in our study should be viewed in light of the all-comer patient sample, as compare in a reasonably favorable fashion to other long-term reports from observational real-world registries on other TAVI devices [29]. Notably, while not-negligible, rates of permanent pacemaker implantation were reasonably low, especially in light of the patient risk profile (mean age of > 82 years, and features of frailty in almost 40% of patients). It is plausible that technical refinements, such as use of the right-left cusp overlap view for TAVI deployment, may further reduce this risk [30]. In addition, and in keeping with other pragmatic studies on patients undergoing TAVI, we found evidence of a constant accrual of adverse events well after the index procedure. Individual risk factors such as peripheral artery disease, chronic obstuctive pulmonary disease, history of atrial fibrillation, prior pacemaker or implantable cardioverter–efibrillator implantation, and depressed systolic function were most impactful prognostically. Such differences in baseline features may at least in part explain discrepant outcomes according to the device size.

Portico and its follower, Navitor, have some distinct advantage especially given the trend toward lower risk patients, including the intra-annular seating, the large cells which can simplify access for subsequent percutaneous coronary revascularization or repeat TAVI, and the dedicated skirt (with Navitor) to minimize leak in patients with extensive calcifications.

### Drawbacks of the present study

Despite the novely of focusing on long-term outcome, several limitations should be considered. First, this is a retrospective observational study with participation of high-volume and established-expertise institutions. Accordingly, the results should not be extrapolated without thought to centers without such experience with TAVI in general, and the Portico valve in particular. Similarly, participating centers contributed on a voluntary basis and are not necessarily representative of the use of the device elsewhere. Most importantly, this is not a randomized or controlled trial, and thus it cannot inform on the comparative effectiveness or safety of Portico when other TAVI devices are also considered as suitable alternatives, nor as a direct proof that Portico can be equivalent or superior to cardiac surgery. Furthermore, the newest iteration of Abbott Vascular TAVI system, the Navitor valve, is now routinely used and includes a sealing skirt. While results of this work can be reasonably applied when informing decision-making on Navitor, dedicated studies on this device are warranted, including long-term follow-up.

## Conclusions

In summary, TAVI with the Portico valve, when performed in selected patients and by experienced structural heart teams, appears associated with favorable clinical outcomes over long-term (> 3 years) follow-up, with few cases of valve deterioration or infection. Clinical outcomes were largely impacted by baseline risk factors and surgical risk, whereas valve size did not impact independently on clinical or imaging endpoints.

### Supplementary Information

Below is the link to the electronic supplementary material.

Supplementary file1 (DOCX 443 KB)

## Declarations

### Data availability statement

The datasets used and/or analysed during the current study available from the corresponding author on reasonable request.
