# Supplementary material for: Long-term outcomes following transcatheter aortic valve implantation with the Portico self-expanding valve
Source: Clin Res Cardiol. 2023 Jun 30;113(1):86–93. doi: 10.1007/s00392-023-02252-x (PMC10808416; doi:10.1007/s00392-023-02252-x)
Supplement: Supplementary file 1 — Supplementary file1 (DOCX 443 KB) [file 392_2023_2252_MOESM1_ESM.docx]

**Long-term outcomes following transcatheter aortic valve implantation with the Portico self-expanding valve**

**Online supplement**

**Table 1S.** Cases per year.

| **Year** | **Patients** |
| --- | --- |
| 2013 | 1 |
| 2014 | 31 |
| 2015 | 69 |
| 2016 | 219 |
| 2017 | 235 |
| 2018 | 176 |
| 2019 | 72 |

**Table 2S.** Cases per participating center.

| **Center** | **Patients** |
| --- | --- |
| A.O.U. Policlinico "G. Rodolico - San Marco", Catania, Italy | 27 |
| Goethe University Hospital, Frankfurt, Germany | 227 |
| Hospital de Santa Cruz, Carnaxide, Portugal | 70 |
| IRCCS San Raffaele Scientific Institute, Milan, Italy | 79 |
| Pineta Grande Hospital, Castel Volturno, Italy | 223 |
| Rigshospitalet, Copenhagen, Denmark | 58 |
| University Heart and Vascular Center Hamburg, Hamburg, Germany | 119 |

**Table 3S.** Baseline imaging features.

| **Feature** | **Count or mean** | **% or standard deviation** |
| --- | --- | --- |
| Patients | 803 | - |
| Left ventricular ejection fraction | 53.0 | 10.8 |
| Aortic stenosis severity |  |  |
| None | 1 | 0.2% |
| Mild | 30 | 5.8% |
| Moderate | 8 | 1.5% |
| Moderate/severe | 227 | 43.5% |
| Severe | 256 | 49.0% |
| Peak aortic valve gradient | 72.3 | 25.8 |
| Mean aortic valve gradient (mm Hg) | 47.2 | 16.7 |
| Aortic valve area (cm^2^) | 0.71 | 0.19 |
| Aortic regurgitation |  |  |
| None | 242 | 31.3% |
| Mild | 369 | 47.7% |
| Moderate | 130 | 16.8% |
| Moderate/severe | 26 | 3.4% |
| Severe | 6 | 0.8% |
| Mitral regurgitation |  |  |
| None | 42 | 5.7% |
| Mild | 401 | 54.7% |
| Moderate | 218 | 29.7% |
| Moderate/severe | 56 | 7.6% |
| Severe | 16 | 2.2% |
| Tricuspid regurgitation |  |  |
| None | 97 | 19.6% |
| Mild | 288 | 58.1% |
| Moderate | 78 | 15.7% |
| Moderate/severe | 29 | 5.9% |
| Severe | 4 | 0.8% |
| Tricuspid annular plane excursion (mm) | 20.5 | 5.1 |
| Systolic pulmonary artery pressure (mm Hg) | 42.4 | 13.7 |

**Table 4S.** Causes of death according to follow-up (p<0.001 at Fisher exact test).

| **Time since transcatheter aortic valve implantation** | **Non-cardiovascular (N=92)** | **Cardiovascular (N=190)** |
| --- | --- | --- |
| ≤1 year (N=86) | 16 (17.4%) | 70 (36.8%) |
| >1 to ≤2 years (N=53) | 8 (8.7%) | 45 (23.7%) |
| >2 to ≤3 years (N=66) | 37 (40.2%) | 29 (15.3%) |
| >3 to ≤4 years (N=30)) | 10 (10.9%) | 20 (10.5%) |
| >4 to ≤5 years (N=21) | 8 (8.7%) | 13 (6.8%) |
| >5 to ≤6 years (N=20) | 8 (8.7%) | 12 (6.3%) |
| >6 to ≤7 years (N=4) | 3 (3.3%) | 1 (0.5%) |
| >7 to ≤8 years (N=2) | 2 (2.2%) | 0 |

**Table 5S.** Long-term imaging features.

| **Feature/outcome** | **Count or mean** | **% or standard deviation** |
| --- | --- | --- |
| Patients | 803 | - |
| Follow-up (years) | 3.1 | 1.5 |
| Left ventricular ejection fraction (%) | 53.7 | 10.6 |
| Structural valve degeneration | 10 | 1.3% |
| Aortic stenosis severity |  |  |
| None | 350 | 91.2% |
| Mild | 31 | 8.1% |
| Moderate | 2 | 0.5% |
| Moderate/severe | 0 | 0 |
| Severe | 1 | 0.3% |
| Peak aortic valve gradient (mm Hg) | 15.2 | 8.2 |
| Mean aortic valve gradient (mm Hg) | 8.1 | 4.6 |
| Patient prosthesis mismatch | 203 | 25.3% |
| Aortic regurgitation |  |  |
| None | 195 | 47.8% |
| Mild | 176 | 43.1% |
| Moderate | 33 | 8.1% |
| Moderate/severe | 3 | 0.7% |
| Severe | 1 | 0.3% |
| Paravalvular leak |  |  |
| None | 141 | 32.6% |
| Mild | 250 | 57.9% |
| Moderate | 37 | 8.6% |
| Moderate/severe | 3 | 0.7% |
| Severe | 1 | 0.2% |
| Mitral regurgitation |  |  |
| None | 63 | 16.6% |
| Mild | 216 | 57.0% |
| Moderate | 76 | 20.1% |
| Moderate/severe | 20 | 5.3% |
| Severe | 4 | 1.1% |
| Tricuspid regurgitation |  |  |
| None | 53 | 14.9% |
| Mild | 221 | 61.9% |
| Moderate | 57 | 16.0% |
| Moderate/severe | 18 | 5.0% |
| Severe | 8 | 2.2% |
| Tricuspid annular plane excursion (mm) | 20.5 | 5.1 |
| Systolic pulmonary artery pressure (mm Hg) | 39.9 | 12.4 |

**Table 6S.** Valve hemodynamic parameters at discharge, overall and according to Portico size.

| **Feature/outcome** | **23** | **25** | **27** | **29** | **Total** | **P** |
| --- | --- | --- | --- | --- | --- | --- |
| Peak aortic valve gradient (mm Hg) | 20.1±10.4 | 15.2±6.1 | 15.6±5.7 | 13.2±6.2 | 15.2±6.7 | <0.001 |
| Mean aortic valve gradient (mm Hg) | 10.8±5.9 | 8.1±3.6 | 8.1±3.2 | 7.2±3.1 | 8.1±3.8 | <0.001 |
| Patient prosthesis mismatch* | 18 (39.1%) | 29 (18.8%) | 35 (20.0%) | 16 (12.0%) | 98 (19.3%) | 0.002 |
| Aortic regurgitation |  |  |  |  |  | <0.001 |
| None | 23 (56.1%) | 62 (45.6%) | 106 (72.1%) | 64 (57.1%) | 255 (58.5%) |  |
| Mild | 16 (39.0%) | 59 (43.4%) | 37 (25.2%) | 33 (29.5%) | 145 (33.3%) |  |
| Moderate | 1 (2.4%) | 14 (10.3%) | 4 (2.7%) | 13 (11.6%) | 32 (7.3%) |  |
| Moderate/severe | 1 (2.4%) | 1 (0.7%) | 0 | 2 (1.8%) | 4 (0.9%) |  |
| Severe | 0 | 0 | 0 | 0 | 0 |  |
| Paravalvular leak |  |  |  |  |  | 0.046 |
| None | 23 (59.0%) | 101 (73.7%) | 110 (71.4%) | 72 (60.0%) | 306 (68.0%) |  |
| Mild | 15 (38.5%) | 31 (22.6%) | 42 (27.3%) | 47 (39.2%) | 135 (30.0%) |  |
| Moderate | 1 (2.6%) | 5 (3.7%) | 2 (1.3%) | 1 (0.8%) | 9 (2.0%) |  |
| Moderate/severe | 0 | 0 | 0 | 0 | 0 |  |
| Severe | 0 | 0 | 0 | 0 | 0 |  |

*based on a mean pressure gradient >10 mm Hg cut-off

**Table 7S.** Valve hemodynamic parameters at long-term follow-up, overall and according to Portico size.

| **Feature/outcome** | **23** | **25** | **27** | **29** | **Total** | **P** |
| --- | --- | --- | --- | --- | --- | --- |
| Peak aortic valve gradient (mm Hg) | 16.1±11.6 | 16.4±12.4 | 14.6±5.9 | 11.8±4.7 | 14.5±9.0 | 0.062 |
| Mean aortic valve gradient (mm Hg) | 8.8±7.5 | 8.6±7.1 | 7.7±3.1 | 6.3±2.4 | 7.7±5.1 | 0.092 |
| Patient prosthesis mismatch* | 18 (39.1%) | 36 (23.4%) | 37 (21.1%) | 17 (12.8%) | 108 (21.3%) | 0.002 |
| Aortic regurgitation |  |  |  |  |  | 0.768 |
| None | 10 (76.9%) | 45 (76.3%) | 42 (84.0%) | 39 (78.0%) | 136 (79.1%) |  |
| Mild | 2 (15.4%) | 12 (20.3%) | 6 (12.0%) | 7 (14.0%) | 27 (15.7%) |  |
| Moderate | 1 (7.7%) | 2 (3.4%) | 2 (4.0%) | 4 (8.0%) | 9 (5.2%) |  |
| Moderate/severe | 0 | 0 | 0 | 0 | 0 |  |
| Severe | 0 | 0 | 0 | 0 | 0 |  |
| Paravalvular leak |  |  |  |  |  | 0.712 |
| None | 3 (23.1%) | 28 (45.2%) | 22 (37.3%) | 20 (34.5%) | 73 (38.0%) |  |
| Mild | 9 (69.2%) | 31 (50.0%) | 33 (55.9%) | 36 (62.1%) | 109 (56.8%) |  |
| Moderate | 1 (7.7%) | 3 (4.8%) | 4 (6.8%) | 2 (3.5%) | 10 (5.2% |  |
| Moderate/severe | 0 | 0 | 0 | 0 | 0 |  |
| Severe | 0 | 0 | 0 | 0 | 0 |  |

*based on a mean pressure gradient >10 mm Hg cut-off

**Table 8S.** Long-term clinical and imaging outcomes according to dichotomized Portico size.

| **Outcome** | **Portico 27 or larger** | **Portico 25 or smaller** | **P** |
| --- | --- | --- | --- |
| Major adverse event* | 76 (24.4%) | 66 (33.7%) | 0.026 |
| Death | 75 (24.0%) | 61 (31.1%) | 0.081 |
| Cardiovascular death | 43 (13.8%) | 34 (17.4%) | 0.310 |
| Stroke | 1 (0.7%) | 4 (5.7%) | 0.042 |
| Myocardial infarction | 0 | 1 (1.4%) | 0.333 |
| Major vascular complication | 15 (10.6%) | 5 (7.1%) | 0.618 |
| Life-threatening bleeding | 2 (1.4%) | 2 (2.0%) | 1 |
| Permanent pacemaker implantation | 36 (24.2%) | 22 (21.3%) | 0.650 |
| Rehospitalization for cardiovascular causes | 16 (11.2%) | 8 (11.9%) | 1 |
| Valve-related dysfunction requiring repeat procedure | 1 (0.7%) | 2 (2.0%) | 0.570 |
| Bioprosthetic valve failure | 74 (23.7%) | 62 (31.6%) | 0.051 |
| Endocarditis | 1 (0.3%) | 0 | 1 |
| Left ventricular ejection fraction (%) | 54.7±8.9 | 55.9±7.1 | 0.333 |
| Aortic stenosis severity |  |  | 0.253 |
| None | 80 (83.3%) | 37 (78.7%) |  |
| Mild | 16 (16.7%) | 8 (17.0%) |  |
| Moderate | 0 | 1 (2.1%) |  |
| Moderate/severe | 0 | 0 |  |
| Severe | 0 | 1 (2.1%) |  |
| Peak aortic valve gradient (mm Hg) | 13.4±5.7 | 16.2±12.2 | 0.045 |
| Mean aortic valve gradient (mm Hg) | 7.2±3.0 | 8.6±7.1 | 0.066 |
| Leaflet thrombosis | 1 (1.0%) | 2 (4.2%) | 0.249 |
| Aortic regurgitation |  |  | 0.460 |
| None | 81 (81.0%) | 55 (76.4%) |  |
| Mild | 13 (13.0%) | 14 (19.4%) |  |
| Moderate | 6 (6.0%) | 3 (4.2%) |  |
| Moderate/severe | 0 | 0 |  |
| Severe | 0 | 0 |  |
| Paravalvular leak |  |  | 0.852 |
| None | 43 (36.4%) | 30 (40.5%) |  |
| Mild | 69 (58.5%) | 40 (54.1%) |  |
| Moderate | 6 (5.1%) | 4 (5.4%) |  |
| Moderate/severe | 0 | 0 |  |
| Severe | 0 | 0 |  |

**Table 9S.** Multivariable predictors of long-term major adverse events or death.*

| **Feature** | **Major adverse cardiac event** | | | **Death** | | |
| --- | --- | --- | --- | --- | --- | --- |
|  | Hazard ratio | 95% confidence interval | P value | Hazard ratio | 95% confidence interval | P value |
| Peripheral artery disease | 1.55 | 1.14-2.09 | 0.005 | 1.56 | 1.15-2.12 | 0.005 |
| Chronic obstructive pulmonary disease | 1.48 | 1.09-2.00 | 0.011 | 1.62 | 1.19-2.20 | 0.002 |
| Estimated glomerular filtration rate | 0.99 | 0.98-1.00 | 0.005 | - | - | - |
| Atrial fibrillation | 1.61 | 1.25-2.08 | <0.001 | 1.91 | 1.49-2.46 | <0.001 |
| Prior pacemaker/implantable cardioverter-defibrillator | 1.46 | 1.01-2.10 | 0.046 | - | - | - |
| EuroSCORE II | 1.03 | 1.02-1.05 | <0.001 | 1.03 | 1.02-1.05 | <0.001 |
| Left ventricular ejection fraction | 0.99 | 0.97-1.00 | 0.009 | 0.98 | 0.97-0.99 | <0.001 |

*models separately built after screening at bivariate analysis the association of each outcome, separately, will variables reported in Tables 1, 2, and 3 (only variables with p<0.05 were entered into the final multivariable model)

**Table 10S.** Life table analysis for long-term major adverse events or death.

| **Interval** | **Beginning total** | | **Cumulative failure rate (95% confidence interval)** | |
| --- | --- | --- | --- | --- |
|  | **Major adverse events** | **Death** | **Major adverse events** | **Death** |
| 0-1 | 803 | 803 | 11.9% (9.8%; 14.3%) | 10.5% (8.6%; 12.8%) |
| 1-2 | 703 | 713 | 16.6% (14.2%; 19.4%) | 16.3% (13.9%; 19.0%) |
| 2-3 | 662 | 664 | 26.5% (23.6%; 29.8%) | 25.0% (22.2%; 28.2%) |
| 3-4 | 575 | 586 | 34.2% (30.8%; 37.9%) | 31.8% (28.5%; 35.4%) |
| 4-5 | 154 | 156 | 44.7% (39.9%; 49.8%) | 42.6% (37.7%; 47.9%) |
| 5-6 | 98 | 99 | 60.2% (53.6%; 66.9%) | 57.9% (51.2%; 64.8%) |
| 6-7 | 31 | 31 | 71.3% (60.1%; 79.9%) | 68.0% (58.6%; 76.9%) |
| 7-8 | 6 | 6 | 79.5% (63.2%; 91.9%) | 77.1% (59.5%; 91.0%) |

**Table 11S.** STROBE Statement—Checklist of items that should be included in reports of cohort studies

|  | Item | Recommendation | Page |
| --- | --- | --- | --- |
| **Title and abstract** | 1 | (*a*) Indicate the study’s design with a commonly used term in the title or the abstract | 1 |
|  |  | (*b*) Provide in the abstract an informative and balanced summary of what was done and what was found | 2 |
| Introduction | | |  |
| Background/rationale | 2 | Explain the scientific background and rationale for the investigation being reported | 4 |
| Objectives | 3 | State specific objectives, including any prespecified hypotheses | 4 |
| Methods | | |  |
| Study design | 4 | Present key elements of study design early in the paper | 5 |
| Setting | 5 | Describe the setting, locations, and relevant dates, including periods of recruitment, exposure, follow-up, and data collection | 5 |
| Participants | 6 | (*a*) Give the eligibility criteria, and the sources and methods of selection of participants. Describe methods of follow-up | 5 |
|  |  | (*b*) For matched studies, give matching criteria and number of exposed and unexposed | NA |
| Variables | 7 | Clearly define all outcomes, exposures, predictors, potential confounders, and effect modifiers. Give diagnostic criteria, if applicable | 5-6 |
| Data sources/ measurement | 8* | For each variable of interest, give sources of data and details of methods of assessment (measurement). Describe comparability of assessment methods if there is more than one group | *5-6* |
| Bias | 9 | Describe any efforts to address potential sources of bias | 5-6 |
| Study size | 10 | Explain how the study size was arrived at | NA |
| Quantitative variables | 11 | Explain how quantitative variables were handled in the analyses. If applicable, describe which groupings were chosen and why | 5-6 |
| Statistical methods | 12 | (*a*) Describe all statistical methods, including those used to control for confounding | 6 |
|  |  | (*b*) Describe any methods used to examine subgroups and interactions | 6 |
|  |  | (*c*) Explain how missing data were addressed | 6 |
|  |  | (*d*) If applicable, explain how loss to follow-up was addressed | 6 |
|  |  | (*e*) Describe any sensitivity analyses | 6 |
| Results | | |  |
| Participants | 13* | (a) Report numbers of individuals at each stage of study—eg numbers potentially eligible, examined for eligibility, confirmed eligible, included in the study, completing follow-up, and analysed | 7-8 |
|  |  | (b) Give reasons for non-participation at each stage | 7-8 |
|  |  | (c) Consider use of a flow diagram | 7-8 |
| Descriptive data | 14* | (a) Give characteristics of study participants (eg demographic, clinical, social) and information on exposures and potential confounders | 7-8 |
|  |  | (b) Indicate number of participants with missing data for each variable of interest | 7-8 |
|  |  | (c) Summarise follow-up time (eg, average and total amount) | 7-8 |
| Outcome data | 15* | Report numbers of outcome events or summary measures over time | 7-8 |
| Main results | 16 | (*a*) Give unadjusted estimates and, if applicable, confounder-adjusted estimates and their precision (eg, 95% confidence interval). Make clear which confounders were adjusted for and why they were included | 7-8 |
|  |  | (*b*) Report category boundaries when continuous variables were categorized | 7-8 |
|  |  | (*c*) If relevant, consider translating estimates of relative risk into absolute risk for a meaningful time period | 7-8 |
| Other analyses | 17 | Report other analyses done—eg analyses of subgroups and interactions, and sensitivity analyses | 8 |
| Discussion | | | 9-12 |
| Key results | 18 | Summarise key results with reference to study objectives | 9-10 |
| Limitations | 19 | Discuss limitations of the study, taking into account sources of potential bias or imprecision. Discuss both direction and magnitude of any potential bias | 11 |
| Interpretation | 20 | Give a cautious overall interpretation of results considering objectives, limitations, multiplicity of analyses, results from similar studies, and other relevant evidence | 9-11 |
| Generalisability | 21 | Discuss the generalisability (external validity) of the study results | 12 |
| Other information | | |  |
| Funding | 22 | Give the source of funding and the role of the funders for the present study and, if applicable, for the original study on which the present article is based | 13 |

**Figure 1S.** Incidence of clinical outcomes during long-term follow-up after transcatheter aortic valve implantation with the Portico valve.

**Figure 2S.** Incidence of major adverse event and death during long-term follow-up after transcatheter aortic valve implantation with the Portico valve according to tertiles of age (Tertile 1: 79 years or less; Tertile 2: 80 to 84 years; Tertile 3: 85 years or more).

|  |
| --- |
|  |
